# Onset and recurrence of psychiatric disorders associated with anti-hypertensive drug classes

**DOI:** 10.1038/s41398-021-01444-1

**Published:** 2021-05-26

**Authors:** Lucy Colbourne, Sierra Luciano, Paul J. Harrison

**Affiliations:** 1 Department of Psychiatry, University of Oxford, Warneford Hospital, Oxford, OX3 7JX UK; 2 Oxford Health NHS Foundation Trust, Oxford, OX3 7JX UK; 3 TriNetX Inc., Cambridge, MA USA

**Keywords:** Bipolar disorder, Depression

## Abstract

The major anti-hypertensive (AHT) drug classes have been associated with differential risks of psychiatric disorders. However, existing data are limited largely to depression, and confounding variables have not always been controlled for. We sought to fill the evidence gap, using TriNetX Analytics, an electronic health records network. Amongst 58.6 million patients aged 18–90 years, patients prescribed a calcium channel blocker (CCB) were compared with those taking a diuretic, angiotensin-converting enzyme inhibitor (ACEI), angiotensin receptor blocker (ARB), or β-blocker. Cohorts were propensity score-matched for age, sex, race, and blood pressure. Over a 2-year exposure period, we measured the incidence and risk ratio of a first diagnosis (ICD-10 codes), or a recurrence, of psychotic, affective, and anxiety disorders, as well as substance use disorders and sleep disorders. Cohort sizes ranged from 33,734 to 322,814. CCBs were associated with a lower incidence of psychotic, affective, and anxiety disorders than β-blockers (risk ratios 0.69–0.99) and a higher incidence than ARBs (risk ratios 1.04–2.23) for both first and recurrent diagnoses. Comparisons of CCBs with ACEIs or diuretics showed smaller risk ratios that varied between disorders, and between first episode and recurrence. AHT classes were also associated with the incidence of substance use and sleep disorders. Results remained largely unchanged after more extensive cohort matching for additional potential confounders. In a secondary analysis, a comparison between ARBs and ACEIs showed lower rates of psychotic, affective, and substance use disorders with ARBs, but higher risks of anxiety and sleep disorders. In conclusion, AHT classes are differentially associated with the incidence of psychiatric disorders. ARBs show the most advantageous profile and β-blockers the least. The apparent beneficial effects of ARBs merit further study.

## Introduction

Anti-hypertensives (AHTs) are widely used to treat high blood pressure and some other cardiovascular indications. There has been interest in the possibility that these drugs may affect the risk for, or recurrence of, depression and other psychiatric disorders, and that these effects may differ between the major classes of AHT: calcium channel blockers (CCBs), diuretics, angiotensin-converting enzyme inhibitors (ACEIs), angiotensin II inhibitors/angiotensin receptor blockers (ARBs), and β-blockers. Given the widespread usage of AHTs, any such differential impacts of AHT classes on mental health could be of considerable public health significance.

To date, the evidence is suggestive but limited. In a Scottish hospital database, the lowest risks of admission for depressive illness over a 5-year period occurred in patients treated with ARBs or ACEIs compared to other AHTs^1^. In a subsequent much larger study, Shaw and colleagues^2^ showed that monotherapy with ARBs or ACEIs was associated with lower rates of a first diagnosis of depression compared to other AHTs, with β-blockers being associated with the highest risk. These authors also found that a diagnosis of bipolar disorder was less common with ARBs and ACEIs. In a retrospective cohort study of hypertensive patients using a Chinese insurance database, lower rates of depression occurred in patients treated with ARBs than with other AHTs; ACEIs and β-blockers were associated with the highest rates^3^. In the Swedish population, patients with serious mental illness, mostly schizophrenia, and bipolar disorder had lower rates of psychiatric hospitalization and self-harm when they were taking CCBs compared to when they were not; other AHTs were not analyzed^4^. Using the Danish population case register, Kessing and colleagues found a reduced incidence of depression^5^ and bipolar disorder^6^ in patients taking ARBs or ACEIs over a 10-year period. In the same population, these authors then showed that the use of all AHT classes except diuretics was associated with a decreased incidence of depression, and reported the novel observation that these effects were observed by some but not all drugs within each class^7^. Agustini et al. studied hypertensive elderly people and found the highest rate of depressive symptoms in those treated with β-blockers compared to other AHTs^8^.

A limitation of the existing data, apart from the heterogeneity of designs and the restriction of most studies to depression, is confounding by indication. That is, differences in outcomes between groups may reflect the non-random allocation of patients to AHT classes and thereby result from differences in their demographic and other characteristics (e.g., age, sex, race), rather than being directly related to the properties of the drugs being compared. Moreover, blood pressure is not always matched between groups or is not reported, and hence it is difficult to disentangle the effects of hypertension and these other variables on psychiatric disorders from those of AHT drugs^9^.

In this study, we attempted to acquire stronger evidence regarding the relationship between AHT classes and psychiatric disorders using an electronic health records network that is large and which allows good control over confounding. In contrast to prior studies, we examined all the major categories of adult psychiatric disorder and examined separately how the drugs affect the incidence of a first diagnosis, and recurrence in those who had a psychiatric diagnosis prior to their AHT exposure.

## Materials and methods

### The TriNetX electronic health records network

TriNetX (www.trinetx.com) is a federated cloud-based network providing access to EHRs from multiple healthcare organizations comprising a mix of hospitals, primary care, and specialty treatment providers. About two-thirds are academic health centers. Aggregated patient data and results from statistical analyses are provided through a browser-based interface. The identity of the health care organizations and the contribution of each one to the cohorts studied is not revealed to researchers, as part of the ethical and legal governance arrangements. The present study used the TriNetX Analytics network, accessing data from ~58.6 million patients aged 18–90 years in 54 health care organizations in the USA. The available data include extensive information on demographics, diagnoses (using International Classification of Diseases, tenth edition [ICD-10] codes), medications, procedures, and lab values. Most data were collected from 2007 onwards. For further details about the network, see Taquet et al.^10^. Our study followed STROBE reporting guidelines (Supplementary Table 1)^11^.

### Creation and matching of cohorts

TriNetX Analytics allows patient cohorts to be created based on defined inclusion and exclusion criteria. Combinations of criteria can be used and with specified temporal relationships between them. For this study, the basic design was to create cohorts of patients exposed to CCBs for the first time and compare their outcomes to cohorts exposed to diuretics, ARBs, ACEIs, or β-blockers, also for the first time. CCBs were chosen as the reference class because of (a) the data of Hayes et al.^4^, (b) the potential repurposing of these drugs for treating psychiatric disorders^12^, and (c) the role of voltage-gated calcium channels in their genetic pathyophysiology^13,14^.

Subjects were aged 18–90 years old at index, were free of any history of “organic” psychiatric disorder (F01–F09, including dementia [F01–F03] and delirium [F05]), and had no prior exposure to the AHTs concerned. Within this eligible study population (58.6 million people) we formed cohorts of two types. In the first, we excluded anyone with a prior psychiatric diagnosis (F20–F48, comprising F20–F29 [psychotic disorders], F30–F39 [affective disorders], and F40–F48 [anxiety disorders]), in order to study the impact of AHTs on the first occurrence of these diagnoses. For the second type of cohort, we only included people who *did* have a prior F20–F48 diagnosis, in order to investigate the effect of AHTs on recurrence (i.e., a further episode of the prior diagnosis, or a different diagnosis within F20–F48). In both types of a cohort, we measured outcomes over a 2-year period. Exposure during this time was proxied by requiring patients to have had two or more prescriptions for the AHT class (though not necessarily the same drug within the class) that were separated by at least a 2-year interval.

As noted, pharmacoepidemiological studies of AHTs can be confounded by differences in blood pressure, and by confounding by indication—in particular, age, sex, and race can all influence the choice of AHT class based on clinical practice guidelines^15,16^. Indeed, we found significant cohort differences in one or more of these variables for all comparisons between CCBs and other AHT classes (data not shown). Hence, for our primary analyses, we matched cohorts at baseline for all these variables. There is also a range of factors that may influence the risk of psychiatric disorders, and which might differ between cohorts, and hence we repeated the analyses with the following variables also matched between cohorts: body mass index, a history of substance misuse disorder, diabetes mellitus or thyroid disease, and prior exposure to AHTs, antidepressants, anxiolytics, antipsychotics, gabapentinoids, opioids, metformin, and statins; for the “recurrence” analyses, we also matched for prior incidence of any F20–F48 diagnosis.

Matching was carried out using the built-in capability to propensity score match cohorts for any variables of interest^17^. TriNetX uses greedy nearest neighbor matching with a caliper distance of 0.1 pooled standard deviations of the logit of the propensity score, to produce 1:1 matching. For further details see Taquet et al.^10^. A standard difference between groups of 0.1 or less is considered negligible^18^.

A list of all ICD-10 codes used is given in Supplementary Table 2.

### Outcomes

The primary outcomes of interest were a diagnosis of a psychotic disorder (F20–F29), mood disorder (F30–F39), or an anxiety disorder (F40–F48) during the 2-year period. We also measured depression (combining F32 [depressive episode] and F33 [recurrent depressive disorder]), as well as substance use disorder (F10–F18), and sleep disorder (F51 or G47). For the “recurrence” analyses, we also measured schizophrenia (F20) and bipolar disorder including manic episode (F30 or F31); we did not measure these disorders in the “first onset” analyses because of their very low incidence (reflecting the age of the cohorts at baseline). In addition, 12 negative control outcomes were measured (Supplementary Table 2)^19^. These are outcomes that have no known nor predicted associations with AHTs and serve as controls for general health and health care usage^20^.

### Data analysis

We used risk ratios, with 95% confidence intervals, to compare outcomes for CCBs relative to each other AHT class. Statistical analyses were performed within the TriNetX Analytics network.

## Results

Using the electronic health records of over 58 million adults in the USA we created cohorts, matched for age, sex, race, and blood pressure, who were then exposed to CCBs or one of the other major AHT classes (diuretics, ACEIs, ARBs, or β-blockers). We measured the risk ratio for onset, or recurrence, of the major psychiatric disorders, as well as sleep and substance abuse disorders, over the following 2 years.

### AHT use and the first diagnosis of psychiatric disorders

In these analyses, all patients were free of any recorded psychiatric disorder (F20–F48) before their first prescription of the AHT class.

The demographic details of each cohort pair, matched for age, sex, race, and blood pressure, are shown in Table 1. The matching was successful for all these variables, except blood pressure in the CCB vs β-blocker comparison, which remained slightly higher in the CCB group (see Table 1 legend). Cohort sizes ranged from 192,161 for CCB vs diuretic, to 270,326 for CCB vs ACEI.

**Table 1:** Baseline cohort demographics for patients without a prior psychiatric (F20–F48) diagnosis.

	CCB	Diuretic	CCB	ACEI	CCB	ARB	CCB	BB
Number	192,161	192,161	270,326	270,326	192,164	192,164	206,703	206,703
Age at index (years)	59.9 (14.7)	59.9 (14.6)	60.5 (14.4)	60.4 (13.8)	60.0 (13.6)	60.3 (12.8)	59.6 (14.3)	59.8 (14.4)
Sex (M:F %)	52: 48	52: 48	48: 52	48: 52	47: 53	46: 54	48: 52	48: 52
Race^a^ (% W,B, O)	64, 21, 15	64, 20, 16	67, 18, 15	67, 19, 14	70, 12, 18	70, 11, 19	66, 21, 13	66, 21, 13
Blood pressure	137/79	136/79	136/78	135/79	133/77	133/78	138/80^b^	134/78^b^
Prior diuretic (%)	–	–	36	37	36	47	34	34
Prior ACEI (%)	29	36	–	–	42	18	31	29
Prior ARB (%)	14	19	32	2	–	–	19	12
Prior BB (%)	30	33	35	28	38	31	–	–
Prior other AHT (%)	10	7	11	6	12	7	8	7
Prior antidepressant (%)	15	14	16	14	18	17	15	17
Prior anxiolytic (%)	20	19	23	17	25	20	18	23
Prior antipsychotic (%)	2	1	2	1	2	1	1	2

Each cohort pair is propensity score-matched for age, sex, race, and blood pressure (most recent recorded value).

^a^W: white, B: black or African American, O: other or not known.

All variables matched except: ^b^SD = 0.19 systolic, 0.16 diastolic.

Results are summarized in Table 2. For CCBs vs diuretics, there is no consistent pattern, and risk ratios are close to one for all major diagnostic categories, except for sleep disorder (RR 0.79 [0.78–0.81]). Compared to ACEIs, CCBs are associated with increased incidence of affective, anxiety, and sleep disorders (RRs 1.05–1.18), and a marginally lower incidence of substance use disorders (RR 0.95 [0.94–0.97]). Compared to ARBs, CCBs are associated with a raised incidence of all diagnoses, especially psychotic disorders (RR 2.23 [2.05–2.45]) and substance abuse disorder (RR 1.94 [1.90–1.99]). In contrast, patients on CCBs have a lower incidence of all diagnoses compared to β-blockers (RRs 0.69–0.97).

**Table 2:** Diagnostic outcomes for patients without a prior psychiatric (F20–F48) diagnosis.

Disorder	ICD-10 codes	CCB vs diuretic		CCB vs ACEI		CCB vs ARB		CCB vs BB	
		% in each cohort	Risk ratio (95% CI)	% in each cohort	Risk ratio (95% CI)	% in each cohort	Risk ratio (95% CI)	% in each cohort	Risk ratio (95% CI)
Psychotic disorder	F20–29	0.6, 0.6	1.02 (0.94–1.11)	0.6, 0.6	1.04 (0.98–1.12)	0.73, 0.33	**2.23 (2.03–2.45)**	0.52, 0.76	**0.69 (0.64–0.75)**
Affective disorder	F30–39	8.3, 8.7	**0.95 (0.93–0.97)**	9.1, 8.7	**1.05 (1.04–1.07)**	9.8, 7.7	**1.27 (1.24–1.29)**	8.6, 9.7	**0.88 (0.86–0.90)**
Depression	F32,33	7.4, 7.8	**0.95 (0.93–0.97)**	8.2, 7.7	**1.06 (1.04–1.08)**	8.8, 7.0	**1.26 (1.23–1.29)**	7.6, 8.6	**0.88 (0.86–0.90)**
Anxiety disorder	F40–48	9.1, 8.6	**1.06 (1.04–1.08)**	9.7, 8.6	**1.13 (1.11–1.15)**	10, 8.6	**1.19 (1.17–1.22)**	9.1, 10	**0.89 (0.87–0.91)**
Sleep disorder	F51, G47	12, 15	**0.79 (0.78–0.81)**	15, 13	**1.18 (1.16–1.19)**	14, 14	1.01 (0.99–1.02)	13, 14	**0.97 (0.95–0.98)**
Substance use disorder	F10–19	9.6, 9.2	**1.04 (1.02–1.06)**	8.7, 9.1	**0.95 (0.94–0.97)**	11, 5.6	**1.94 (1.90–1.99)**	8.7, 10	**0.86 (0.84–0.87)**
Negative control outcomes^a^		–	**0.88 (0.82–0.93)**	–	0.96 (0.90–1.02)	–	1.00 (0.93–1.08)	–	**1.11 (1.02–1.19)**

^a^Mean of 12 negative control outcomes (see Supplementary Table 1).

Bold values indicate statistical significance.

### AHT use and the recurrence of psychiatric disorders

In these analyses, all patients had a prior psychiatric disorder (F20–F48), and we measured the incidence of a further such diagnosis during the 2 years’ prescription of the AHTs concerned.

The demographic details of each pair of cohorts, matched for age, sex, race, and blood pressure, are shown in Table 3. The matching was again successful for all variables except blood pressure in the CCB vs β-blocker comparison (see Table 3 legend). Cohort sizes ranged from 33,734 for CCBs vs diuretics, to 48,590 for CCBs vs ACEIs.

**Table 3:** Baseline cohort demographics for patients with a prior psychiatric (F20–F48) diagnosis.

	CCB	Diuretic	CCB	ACEI	CCB	ARB	CCB	BB
Number	33,734	33,734	48,590	48,590	37,229	37,229	39,281	39,281
Age at index (years)	56.4 (15.0)	56.5 (14.7)	57.3 (14.8)	57.1 (13.3)	57.8 (14.1)	58.0 (12.7)	56.7 (14.2)	57.1 (14.4)
Sex (M:F %)	39: 61	38: 62	33: 67	33: 67	30: 70	31: 69	34: 66	34: 66
Race (% W, B, O)^b^	77, 17, 6	76, 17, 7	79, 16, 5	79, 15, 6	84, 10, 6	83, 10, 7	75, 19, 6	75, 19, 6
Blood pressure	134/79	132/78	134/78	134/79	133/77	132/78	136/80^a^	130/76^b^
Prior diuretic (%)	–	–	41	42	44	51	41	36
Prior ACEI (%)	30	34	–	–	44	35	36	28
Prior ARB (%)	12	15	28	4	–	–	18	11
Prior BB (%)	35	33	40	31	44	35	–	–
Prior other AHT (%)	14	10	16	10	16	10	12	10
Prior F20–29 (%)	6	5	6	6	6	3	6	7
Prior F30–39 (%)	65	67	66	67	68	66	67	66
Prior F40–48 (%)	64	62	64	61	63	63	62	64
Prior F10–19 (%)	25	24	24	25	28	19	25	24
Prior antidepressant (%)	59	57	60	59	63	64	60	58
Prior anxiolytic (%)	53	49	55	48	57	52	49	53
Prior antipsychotic (%)	12	10	12	10	12	7	10	12

Cohorts propensity score-matched for age, sex, race, and blood pressure (most recent recorded value). “Prior” means any occurrence before the start of the exposure period.

^a^Blood pressure could not be matched for CCB vs BB (SD = 0.31 for systolic and diastolic).

^b^W: white. B: black or African American. O: other or not known.

Results are summarized in Table 4. Most cohort comparisons show similar findings as for the first onset analyses. That is, the incidence of recurrent disorders is higher with CCBs than ARBs but lower with CCBs than β-blockers, and with no consistent findings for CCBs vs diuretics other than for sleep disorder. For CCBs vs ACEIs, there were few differences, but there was a lower risk of recurrence for psychotic disorders (RR 0.91 [0.86–0.97]), and a slight increase in the incidence of anxiety disorders (RR 1.05 [1.04–1.07]).

**Table 4:** Diagnostic outcomes for patients with a prior psychiatric (F20–F48) diagnosis.

Disorder	ICD-10 codes	CCB vs Diuretic		CCB vs ACEI		CCB vs ARB		CCB vs BB	
		% in each cohort	Risk ratio (95% CI)	% in each cohort	Risk ratio (95% CI)	% in each cohort	Risk ratio (95% CI)	% in each cohort	Risk ratio (95% CI)
Psychotic disorder	F20–29	4.1, 3.8	1.07 (0.99–1.15)	3.7, 4.0	**0.91 (0.86–0.97)**	3.8, 2.1	**1.82 (1.67–1.98)**	3.6, 4.7	**0.76 (0.71–0.81)**
Schizophrenia	F20	2.0, 1.7	**1.15 (1.03–1.28)**	1.6, 1.9	**0.82 (0.74–0.89)**	1.6, 0.8	**1.95 (1.70–2.24)**	1.7, 2.2	**0.77 (0.70–0.86)**
Affective disorder	F30–39	46, 46	0.99 (0.97–1.01)	46, 46	1.00 (0.99–1.01)	49, 45	**1.08 (1.06–1.09)**	46, 46	0.99 (0.98–1.01)
Bipolar disorder	F30,31	6.1, 5.4	**1.13 (1.07–1.21)**	5.4, 5.5	0.99 (0.94–1.05)	6.2, 3.7	**1.67 (1.56–1.78)**	5.2, 6.1	**0.86 (0.82–0.91)**
Depression	F32,33	39, 40	**0.98 (0.96–0.99)**	41, 41	1.00 (0.99–1.02)	43, 41	**1.06 (1.04–1.08)**	40, 41	0.99 (0.97–1.01)
Anxiety disorder	F40–48	42, 41	**1.04 (1.02–1.05)**	43, 41	**1.05 (1.04–1.07)**	44, 42	**1.04 (1.03–1.06)**	41, 44	**0.94 (0.92–0.95)**
Sleep disorder	F51, G47	26, 29	**0.89 (0.87–0.92)**	31, 27	**1.16 (1.13–1.18)**	31, 31	1.00 (0.98–1.03)	28, 29	0.97 (0.95–1.00)
Substance use disorder	F10–19	21, 20	**1.08 (1.05–1.11)**	19, 20	**0.96 (0.93–0.98)**	22, 14	**1.57 (1.52–1.62)**	19, 20	0.97 (0.95–1.00)
Negative control outcomes^a^		–	**0.88 (0.82–0.94)**	–	1.07 (1.00-1.14)	–	0.99 (0.89–1.10)	–	1.00 (0.92–1.08)

^a^Mean of 12 negative control outcomes (see Supplementary Table 1).

Bold values indicate statistical significance.

### Negative control outcomes

As shown in Tables 2 and 4, there was no difference in the mean of the negative control outcomes between CCBs and ACEIs or ARBs. Negative control outcomes were less common with CCBs than diuretics in both the first onset (RR 0.88 [0.82–0.93]; Table 2) and recurrence (RR 0.88 [0.82–0.94]; Table 4) cohorts. Negative control outcomes were more common with CCBs than β-blockers in those without a prior psychiatric diagnosis (1.11 [1.02–1.19]; Table 2) but not in those with a prior history (1.00 [0.92–1.08]; Table 4).

### Sensitivity and secondary analyses

Results for comparisons made after the more extensive propensity score matching for additional risk factors (as listed in “Materials and methods”) are shown in Supplementary Table 3 (for the first onset of psychiatric disorders) and Supplementary Table 4 (for recurrence). The results are unchanged from those of the primary analyses in terms of significant findings, although relative risks for some outcomes are attenuated.

Finally, because of the greater benefits of ARBs than ACEIs compared to CCBs (Tables 2 and 4), we carried out a secondary analysis in which ARBs were compared directly to ACEIs. Table 5 shows that ARBs are associated with lower risks of psychotic, affective, and substance use disorders, but with higher risks of anxiety and sleep disorders. These effects were seen for both first onset and recurrence.

**Table 5:** Comparison of ARBs with ACEIs.

		ARB vs ACEI (prior psychiatric diagnosis)		ARB vs ACEI (no prior psychiatric diagnosis)	
Baseline characteristics		ARB	ACEI	ARB	ACEI
Number		49,314	49,314	322,814	322,814
Age at index (years)		60.6 (12.8)	60.6 (12.8)	62.3 (12.4)	62.4 (12.6)
Sex (M:F %)		30:70	30:70	46:54	46:54
Race (% W, B, O)^a^		76, 15, 9	77, 15, 8	65, 16, 19	66, 16, 18
Blood pressure		135/78	135/78	137/78	137/78
Outcomes	ICD-10 codes	% in each cohort (ARB, ACEI)	Risk ratio (95% CI)	% in each cohort (ARB, ACEI)	Risk ratio (95% CI)
Psychotic disorder	F20-29	2.5, 4.0	**0.61 (0.57–0.66)**	0.4, 0.7	**0.57 (0.54–0.61)**
Schizophrenia	F20	0.9, 1.8	**0.52 (0.46–0.58)**	0.08, 0.2	**0.39 (0.33–0.45)**
Affective disorder	F30-39	45, 48	**0.94 (0.93–0.95)**	8.7, 9.4	**0.92 (0.91–0.94)**
Bipolar disorder	F30,31	4.0, 5.5	**0.73 (0.69–0.77)**	0.4, 0.6	**0.68 (0.63–0.73)**
Major depression	F32,33	40, 43	**0.94 (0.93–0.96)**	7.9, 8.4	**0.93 (0.92–0.95)**
Anxiety disorder	F40-48	44, 42	**1.03 (1.02–1.05)**	9.2, 8.7	**1.05 (1.03–1.06)**
Sleep disorder	F51, G47	32,29	**1.13 (1.11–1.15)**	16, 13	**1.22 (1.21–1.24)**
Substance use disorder	F10-19	14,21	**0.66 (0.64–0.68)**	5.7, 9.5	**0.60 (0.59–0.61)**

Cohorts propensity score-matched for age, sex, race, and blood pressure (most recent recorded value).

^a^W: white, B: black or African American, O: other or unknown.

Bold values indicate statistical significance.

## Discussion

Prior observational studies have suggested that AHT classes may be associated with a differential incidence of onset or relapse of depression^1–3,5,7,8^ and some other psychiatric disorders^2,4,6^. Our data extend this evidence and clarify its interpretation, by virtue of the much larger sample sizes, the richness of available data, closer control over confounding factors, and the use of negative control outcomes. We also provide the first large-scale data regarding AHTs and anxiety disorders. In brief, our results suggest a hierarchy of effects in which ARBs are associated with the lowest rates of affective, anxiety, and psychotic disorders, and β-blockers the highest; only minor differences are observed between CCBs, ACEIs, and diuretics.

For each cohort pair, the direction and magnitude of the effect were generally similar for all three major diagnostic categories. For example, CCBs had a comparably lower incidence of affective, anxiety, and psychotic disorders compared to β-blockers. On the other hand, there were also some points of difference. In the CCB vs ARB contrast, the risk ratios for the first diagnosis of psychotic disorders (including bipolar disorder) were significantly larger than for affective and anxiety disorders, whereas in the CCB vs diuretic comparison, affective disorders were less common with CCBs but anxiety disorders were more common. The risk ratios for substance misuse disorder and sleep disorder also showed differences between CCBs and the other AHT classes, but the pattern did not show a consistent relationship to the effects on the other diagnostic categories. Interpretation of the sleep disorder data is also complicated by the possibility that the different AHT classes produce different degrees of nocturnal diuresis.

The findings were broadly similar for the first onset and for relapse. Since these cohorts are mutually exclusive, this may be seen as a form of replication. The convergence may also shed some light on possible explanations. For example, the effect on first diagnosis rules out the possibility that results are confounded by differential prescribing of AHTs to patients with a known psychiatric disorder, whilst the effect on recurrence suggests that the mechanism of the AHT effects applies equally to the processes maintaining psychiatric disorders as to their initial triggering.

We previously reported, in this electronic health records network and over the same 2-year exposure period, that AHT classes are associated with a differential incidence of delirium^19^ and of neurodegenerative and cerebrovascular diseases^21^. In those studies, ACEIs and ARBs were grouped together as renin-angiotensin system (RAS) agents. An overview of their results, together with the current findings, are provided in Table 6, which shows that RAS agents are associated with a lower incidence of all outcomes compared to CCBs and, by extrapolation, compared to diuretics and β-blockers. In the current study, where we compared ARBs and ACEIs separately against CCBs, our primary analyses suggested that ARBs might be superior to ACEIs, based on their respective risk ratios compared to CCBs. Supporting this conclusion, we then compared ARBs directly with ACEIs, and showed a lower risk of all outcomes with ARBs, except for sleep disorders and marginally for anxiety disorders (Table 5). These findings extend a range of previous findings indicating a beneficial effect of ARBs on the risk of psychiatric disorders^1–3,5,7,22^, neurodegenerative diseases^23^, and quality of life^24^. Various mechanisms have been postulated, mostly focusing on central angiotensin receptors and their interaction with the dopamine system^25^, and their anti-inflammatory properties^7^. Human and preclinical data support a role for these receptors in stress and anxiety phenotypes^26–29^. The other clear finding in Table 6 is that, with the exception of dementia, β-blockers are associated with the highest risk of all psychiatric outcomes. This result adds to the evidence that this class of drugs may have a deleterious effect on depression and other psychiatric disorders^1–3,8,30^, though see also ref. ^7^

**Table 6:** Comparative effects of 2 years’ exposure to CCBs versus other antihypertensive drug classes on the incidence of the first diagnosis of psychiatric, neurodegenerative, and cerebrovascular disorders. All cohorts were matched for age, sex, race, and blood pressure. Numbers in brackets are risk ratios (this study) or odds ratios (prior studies). “Higher” and “lower” indicate results with confidence intervals not overlapping 1.

	ICD-10 codes	Incidence with CCBs compared to				
		Diuretics	ACEI	ARB	RAS agents	β-blockers
Psychotic disorder	F20–29	Similar (1.02)	Similar (1.04)	Higher (2.23)	–	Lower (0.69)
Affective disorder	F30–39	Lower (0.95)	Higher (1.05)	Higher (1.27)	–	Lower (0.88)
Anxiety disorder	F40–48	Higher (1.06)	Higher (1.13)	Higher (1.19)	–	Lower (0.89)
Sleep disorder	F51, G47	Lower (0.79)	Higher (1.18)	Similar (1.01)	–	Lower (0.97)
Substance abuse disorder	F10–F19	Higher (1.04)	Lower (0.95)	Higher (1.94)	–	Lower (0.86)
Delirium^a,c^	F05; R40.0, R41.0	Lower (0.81)	–	–	Higher (1.51)	Lower (0.78)
Dementia^b,c^	F01–03; G30, G31.0, G31.2, G31.83	Higher (1.19)	–	–	Higher (1.24)	Similar (0.96)
Movement disorder^b,c^	G20–26	Lower (0.92)	–	–	Higher (1.21)	Lower (0.73)
Cerebrovascular disease^b,c^	I60–69	Higher (1.17)	–	–	Higher (1.34)	Lower (0.86)

^a^Data from ref. ^19^.

^b^Data from ref. ^21^.

^c^In these studies, ACEIs, and ARBs were grouped together as renin-angiotensin system (RAS) agents.

### Limitations

Our study has strengths, notably its size, the range of disorders examined, the distinction between first onset and recurrence, and the propensity score matching. However, it also has several limitations. (1) The possibility of confounding by indication cannot be eliminated despite the careful matching of cohorts and the sensitivity analyses^31,32^, and the modest size of many of the risk ratios highlights the need for caution. (2) The completeness and accuracy of EHRs, including the psychiatric diagnoses, is unknown^33–35^. (3) To enhance the generalizability of the findings, we did not control for use of other medications during the exposure period, and these might contribute to, or mitigate against, differences between cohorts. Nor do we know about compliance or drug dosage, nor whether patients switched between drugs within an AHT class; this is relevant given the finding that individual agents within a class may confer differential effects on depression risk^7^. (4) Our design means that we only measured outcomes in patients who were prescribed an AHT class for two years, and so individuals who discontinued medication during this time will have been excluded. (5) There was a lower incidence of the negative control outcomes with CCBs than with diuretics, suggesting either an overall better health profile in patients taking CCBs or that they present less often for health care. Either way, it complicates the interpretation of the primary outcomes, which showed much smaller differences in the incidence of psychiatric diagnoses between CCB and diuretic cohorts. Finally, as with all observational studies, it is not possible to draw strong causal inferences; randomized controlled trials would be required to determine whether AHT classes impact differentially on the risk of psychiatric disorders.

### Repurposing of AHTs for psychiatric disorders

There has been interest in the repurposing of CCBs to treat psychiatric disorders^14^, notably bipolar disorder^12^, based upon the putative involvement of calcium ions in its pathophysiology^36^ and supported more recently by the genomic association of voltage-gated calcium channel subunits across a range of psychiatric disorders^13^. The present results do not provide support for psychiatric repurposing of existing CCBs, and the existing clinical evidence is also weak^12^. However, as a caveat, over 90% of patients in the CCB cohorts were taking amlodipine, verapamil, or diltiazem, which all have low brain penetrability and/or limited channel subtype selectivity^37^. Hence the possibility that more brain penetrant, brain selective, CCBs might be of value for psychiatric indications remains to be tested^38,39^. This could be done in conjunction with the evaluation of ARBs, which have also been proposed to be of use for psychiatric indications^40^. Indeed, as noted above, our data support and extend the emerging evidence that ARBs may have particular benefits for brain health.

In summary, AHT classes are associated with differential risks of psychiatric disorders. The findings are not readily attributable to known confounders. Whilst caution is always required when interpreting observational data, the results suggest that, other things being equal, the choice of AHT class should take into account a patient’s psychiatric history, and should be reviewed if a person develops a psychiatric disorder whilst taking an AHT.

## Supplementary information

Supplemental Material

## Data Availability

Data subject to third-party restrictions. The data are available from the authors with the permission of TriNetX.
