# Supplementary material for: Onset and recurrence of psychiatric disorders associated with anti-hypertensive drug classes
Source: Transl Psychiatry. 2021 May 26;11:319. doi: 10.1038/s41398-021-01444-1 (PMC8155006; doi:10.1038/s41398-021-01444-1)
Supplement: Supplementary file 1 — Supplemental Material [file 41398_2021_1444_MOESM1_ESM.docx]

**Supplementary Tables 1-4 for ‘Onset and recurrence of psychiatric disorders associated with anti-hypertensive drug classes’, by Colbourne, Luciano and Harrison**

Supplementary Table 1 – STROBE checklist

|  | Item No. | Recommendation | Page  No. | Relevant text from manuscript |
| --- | --- | --- | --- | --- |
| **Title and abstract** | 1 | (*a*) Indicate the study’s design with a commonly used term in the title or the abstract | 2 |  |
|  |  | (*b*) Provide in the abstract an informative and balanced summary of what was done and what was found | 2 |  |
| Introduction | | | |  |
| Background/rationale | 2 | Explain the scientific background and rationale for the investigation being reported | 3-4 |  |
| Objectives | 3 | State specific objectives, including any prespecified hypotheses | 4 |  |
| Methods | | | |  |
| Study design | 4 | Present key elements of study design early in the paper | 4-5 |  |
| Setting | 5 | Describe the setting, locations, and relevant dates, including periods of recruitment, exposure, follow-up, and data collection | 4 |  |
| Participants | 6 | (*a*) *Cohort study*—Give the eligibility criteria, and the sources and methods of selection of participants. Describe methods of follow-up  *Case-control study*—Give the eligibility criteria, and the sources and methods of case ascertainment and control selection. Give the rationale for the choice of cases and controls  *Cross-sectional study*—Give the eligibility criteria, and the sources and methods of selection of participants | 4 |  |
|  |  | (*b*) *Cohort study*—For matched studies, give matching criteria and number of exposed and unexposed  *Case-control study*—For matched studies, give matching criteria and the number of controls per case | 4-5 |  |
| Variables | 7 | Clearly define all outcomes, exposures, predictors, potential confounders, and effect modifiers. Give diagnostic criteria, if applicable | 5 |  |
| Data sources/ measurement | 8* | For each variable of interest, give sources of data and details of methods of assessment (measurement). Describe comparability of assessment methods if there is more than one group | 4 |  |
| Bias | 9 | Describe any efforts to address potential sources of bias | 5,9 |  |
| Study size | 10 | Explain how the study size was arrived at | 5 |  |

Continued on next page

| Quantitative variables | 11 | Explain how quantitative variables were handled in the analyses. If applicable, describe which groupings were chosen and why | NA |  | |
| --- | --- | --- | --- | --- | --- |
| Statistical methods | 12 | (*a*) Describe all statistical methods, including those used to control for confounding | 5,6 |  | |
|  |  | (*b*) Describe any methods used to examine subgroups and interactions | 5 |  | |
|  |  | (*c*) Explain how missing data were addressed | NA |  | |
|  |  | (*d*) *Cohort study*—If applicable, explain how loss to follow-up was addressed  *Case-control study*—If applicable, explain how matching of cases and controls was addressed  *Cross-sectional study*—If applicable, describe analytical methods taking account of sampling strategy | NA |  | |
|  |  | (*e*) Describe any sensitivity analyses | 5 |  | |
| Results | | | | | |
| Participants | 13* | (a) Report numbers of individuals at each stage of study—eg numbers potentially eligible, examined for eligibility, confirmed eligible, included in the study, completing follow-up, and analysed | 6-7, Tables 1,3,5 | |  |
|  |  | (b) Give reasons for non-participation at each stage |  | |  |
|  |  | (c) Consider use of a flow diagram |  | |  |
| Descriptive data | 14* | (a) Give characteristics of study participants (eg demographic, clinical, social) and information on exposures and potential confounders | 6-7, Tables 1-5 | |  |
|  |  | (b) Indicate number of participants with missing data for each variable of interest |  | |  |
|  |  | (c) *Cohort study*—Summarise follow-up time (eg, average and total amount) |  | |  |
| Outcome data | 15* | *Cohort study*—Report numbers of outcome events or summary measures over time | 6-7, Tables 2,4,5 | |  |
|  |  | *Case-control study—*Report numbers in each exposure category, or summary measures of exposure |  | |  |
|  |  | *Cross-sectional study—*Report numbers of outcome events or summary measures |  | |  |
| Main results | 16 | (*a*) Give unadjusted estimates and, if applicable, confounder-adjusted estimates and their precision (eg, 95% confidence interval). Make clear which confounders were adjusted for and why they were included | Estimates on pp 6-7 and Tables 2,4,5; fully adjusted estimates in Suppl Tables 3 and 4 | |  |
|  |  | (*b*) Report category boundaries when continuous variables were categorized |  |  | |
|  |  | (*c*) If relevant, consider translating estimates of relative risk into absolute risk for a meaningful time period |  |  | |

Continued on next page

| Other analyses | 17 | Report other analyses done—eg analyses of subgroups and interactions, and sensitivity analyses | p7, Table 5, and Suppl Tables 3 and 4 |  |
| --- | --- | --- | --- | --- |
| Discussion | | | | |
| Key results | 18 | Summarise key results with reference to study objectives | P7-8 |  |
| Limitations | 19 | Discuss limitations of the study, taking into account sources of potential bias or imprecision. Discuss both direction and magnitude of any potential bias | 9 |  |
| Interpretation | 20 | Give a cautious overall interpretation of results considering objectives, limitations, multiplicity of analyses, results from similar studies, and other relevant evidence | 7-8 |  |
| Generalisability | 21 | Discuss the generalisability (external validity) of the study results | 9 |  |
| Other information | |  | | |
| Funding | 22 | Give the source of funding and the role of the funders for the present study and, if applicable, for the original study on which the present article is based | 9 |  |

Supplementary Table 2. ICD-10 diagnostic codes used for outcomes, exclusions, and propensity score matching

| **Category** | **ICD-10 code(s)** | **Main sub-categories** |
| --- | --- | --- |
| ***Diagnoses excluded from all cohorts at baseline*** | | |
| Organic mental disorders | F01-F09 | F00-F03 (dementias), F05 (delirium) |
|  |  |  |
| ***Propensity score matched diagnoses used in sensitivity analysis*** | | |
| Substance abuse disorder | F10-F19 |  |
| Diabetes mellitus | E08-E13 |  |
| Thyroid disorders | E00-E07 |  |
|  |  |  |
| ***Outcomes*** | | |
| Psychotic disorder | F20-F29 | F20 (schizophrenia) |
| Affective disorder | F30-F39 | F30 (manic episode), F31 (bipolar disorder), F32 (depressive episode), F33 (recurrent depressive disorder |
| Anxiety disorder | F40-F48 |  |
| Sleep disorder | F51, G47 | F51 (sleep disorder not due to a substance or physiological condition), G47 (sleep disorders) |
| Substance abuse disorder | F10-F19 |  |
|  | | |
| ***Negative control outcomes*** | | |
| Benign colonic polyp | D12.0 |  |
| Cutaneous abscess | L02 |  |
| Ganglion | M67.4 |  |
| Hallux valgus (acquired) | M20.1 |  |
| Hernia | K40-K46 |  |
| Ingrowing nail | L60.0 |  |
| Onycholysis | L60.1 |  |
| Otalgia | H92.09 |  |
| Sebaceous cyst | L72.3 |  |
| Senile keratosis | L82.1 |  |
| Trigger finger | M65.3 |  |
| Viral warts | B07 |  |

Supplementary Table 3. Diagnostic outcomes after additional propensity score matching, for patients without a prior psychiatric (F20-F48) diagnosis^a^

| Disorder | ICD-10 codes | CCB vs diuretic | |  | CCB vs ACEI | |  | CCB vs ARB | |  | CCB vs BB | |
| --- | --- | --- | --- | --- | --- | --- | --- | --- | --- | --- | --- | --- |
|  |  | % in each cohort | Risk ratio (95% CI) |  | % in each cohort | Risk ratio (95% CI) |  | % in each cohort | Risk ratio (95% CI) |  | % in each cohort | Risk ratio (95% CI) |
| Psychotic disorder | F20-29 | 0.6, 0.6 | 0.96 (0.89-1.04) |  | 0.7, 0.6 | **1.17 (1.08-1.26)** |  | 0.7, 0.3 | **2.17 (1.97-2.39)** |  | 0.5, 0.7 | **0.74 (0.68-0.80)** |
| Affective disorder | F30-39 | 8.3, 8.6 | **0.97 (0.95-0.99)** |  | 9.4, 8.6 | **1.08 (1.06-1.11)** |  | 9.7, 7.8 | **1.24 (1.21-1.27)** |  | 8.8, 9.7 | **0.91 (0.89-0.93)** |
| Major depression | F32,33 | 7.4, 7.7 | **0.96 (0.94-0.98)** |  | 8.4, 7.6 | **1.10 (1.07-1.12)** |  | 8.7, 7.0 | **1.23 (1.21-1.26)** |  | 7.8, 8.6 | **0.91 (0.89-0.93)** |
| Anxiety disorder | F40-48 | 9.2, 8.8 | **1.04 (1.02-1.06)** |  | 10.1, 9.0 | **1.12 (1.10-1.14)** |  | 10, 8.7 | **1.15 (1.12-1.17)** |  | 9.4, 10.4 | **0.90 (0.89-0.92)** |
| Sleep disorder | F51, G47 | 12,14 | **0.82 (0.81-0.84)** |  | 14, 12 | **1.15 (1.13-1.17)** |  | 14, 15 | **0.98 (0.96-0.99)** |  | 14, 13 | 1.01 (1.00-1.03) |
| Substance use disorder | F10-19 | 9.5, 9.6 | 0.98 (0.96-1.00) |  | 9.3, 9.2 | 1.01 (0.99-1.03) |  | 8.8, 6.1 | **1.45 (1.42-1.49)** |  | 8.6, 9.2 | **0.93 (0.91-0.95)** |
|  |  |  |  |  |  |  |  |  |  |  |  |  |
| Negative control outcomes^b^ |  |  | **0.85 (0.82-0.89)** |  |  | 0.99 (0.93-1.04) |  |  | 0.96 (0.88-1.04) |  |  | 1.06 (0.98-1.14) |

^a^For the list of additional variables matched, see text.

^b^Mean of 12 negative control outcomes. See text for details.

Supplementary Table 4. Diagnostic outcomes after additional propensity score matching, for patients with a prior psychiatric (F20-F48) diagnosis^a^

| Disorder | ICD-10 codes | CCB vs Diuretic | |  | CCB vs ACEI | |  | CCB vs ARB | |  | CCB vs BB | |
| --- | --- | --- | --- | --- | --- | --- | --- | --- | --- | --- | --- | --- |
|  |  | % in each cohort | Risk ratio (95% CI) |  | % in each cohort | Risk ratio (95% CI) |  | % in each cohort | Risk ratio (95% CI) |  | % in each cohort | Risk ratio (95% CI) |
| Psychotic disorder | F20-29 | 4.1, 3.7 | **1.10 (1.02-1.18)** |  | 4.0, 3.8 | 1.04 (0.97-1.12) |  | 3.3, 2.2 | **1.51 (1.38-1.67)** |  | 3.5, 4.3 | **0.80 (0.75-0.86)** |
| Schizophrenia | F20 | 2.0, 1.7 | **1.18 (1.06-1.32)** |  | 1.8, 2.4 | 0.79 (0.72-0.86) |  | 1.3, 0.8 | **1.48 (1.28-1.71)** |  | 1.7, 2.0 | **0.84 (0.76-0.94)** |
| Affective disorder | F30-39 | 45, 46 | 0.99 (0.98-1.02) |  | 46, 46 | 1.01 (1.00-1.02) |  | 47, 45 | **1.04 (1.02-1.06)** |  | 46, 46 | 0.99 (0.98-1.01) |
| Bipolar disorder | F30,31 | 6.1, 5.1 | **1.19 (1.12-1.27)** |  | 5.8, 5.4 | **1.08 (1.02-1.14)** |  | 4.9, 3.8 | **1.27 (1.19-1.37)** |  | 5.3, 5.9 | **0.89 (0.84-0.94)** |
| Major depression | F32,33 | 39, 40 | 0.98 (0.96-1.00) |  | 41, 40 | 1.01 (0.99-1.03) |  | 42, 40 | **1.04 (1.02-1.06)** |  | 40, 41 | 0.99 (0.98-1.01) |
| Anxiety disorder | F40-48 | 42, 41 | **1.03 (1.01-1.05)** |  | 43, 42 | 1.02 (1.00-1.04) |  | 42, 42 | 1.01 (0.99-1.02) |  | 41, 44 | **0.93 (0.91-0.94)** |
| Sleep disorder | F51, G47 | 26, 28 | **0.93 (0.91-0.95)** |  | 29, 27 | **1.10 (1.08-1.13)** |  | 30, 31 | 0.98 (0.96-1.01) |  | 28, 29 | **0.97 (0.94-0.99)** |
| Substance use disorder | F10-19 | 21, 21 | 1.02 (0.99-1.05) |  | 20, 21 | **0.95 (0.92-0.98)** |  | 17, 15 | **1.17 (1.13-1.21)** |  | 19, 19 | 0.98 (0.96-1.01) |
|  |  |  |  |  |  |  |  |  |  |  |  |  |
| Negative control outcomes^b^ |  |  | **0.88 (0.83-0.92)** |  |  | **1.09 (1.03-1.15)** |  |  | 0.96 (0.86-1.05) |  |  | 0.98 (0.92-1.05) |

^a^For the list of additional variables matched, see text.

^b^Mean of 12 negative control outcomes. See text for details.
